# Development of a deep learning-based model for guiding a dissection during robotic breast surgery

**DOI:** 10.1186/s13058-025-01981-3

**Published:** 2025-03-10

**Authors:** Jeea Lee, Sungwon Ham, Namkug Kim, Hyung Seok Park

**Affiliations:** 1 Department of Surgery, Uijeongbu Eulji Medical Center, Eulji University, Uijeongbu-si, Gyeonggi-do South Korea; 2 Department of Surgery, Graduate School of Medicine, Yonsei University College of Medicine, Seoul, South Korea; 3 Healthcare Readiness Institute for Unified Korea, Korea University Ansan Hospital, Korea University College of Medicine, 123 Jeokgeum-ro, Danwon-gu, Ansan City, 15355 Gyeonggi-do South Korea; 4 Department of Convergence Medicine, University of Ulsan College of Medicine, Asan Medical Center, Seoul, South Korea; 5 Department of Radiology and Research Institute of Radiology, University of Ulsan College of Medicine, Asan Medical Center, Seoul, South Korea; 6 Department of Surgery, Yonsei University College of Medicine, 50-1, Yonsei-ro, Seodaemun-gu, Seoul, 03722 South Korea; 7 Institute for Innovation in Digital Healthcare, Yonsei University, Seoul, South Korea

**Keywords:** Artificial intelligence, Deep learning, Minimally invasive surgical procedures, Breast neoplasms, Robotic mastectomy

## Abstract

**Background:**

Traditional surgical education is based on observation and assistance in surgical practice. Recently introduced deep learning (DL) techniques enable the recognition of the surgical view and automatic identification of surgical landmarks. However, there was no previous studies have conducted to develop surgical guide for robotic breast surgery. To develop a DL model for guiding the dissection plane during robotic mastectomy for beginners and trainees.

**Methods:**

Ten surgical videos of robotic mastectomy procedures were recorded. Video frames taken at 1-s intervals were converted to PNG format. The ground truth was manually delineated by two experienced surgeons using ImageJ software. The evaluation metrics were the Dice similarity coefficient (DSC) and Hausdorff distance (HD).

**Results:**

A total of 8,834 images were extracted from ten surgical videos of robotic mastectomies performed between 2016 and 2020. Skin flap dissection during the robotic mastectomy console time was recorded. The median age and body mass index of the patients was 47.5 (38–52) years and 22.00 (19.30–29.52) kg/m^2^, respectively, and the median console time was 32 (21–48) min. Among the 8,834 images, 428 were selected and divided into training, validation, and testing datasets at a ratio of 7:1:2. Two experts determined that the DSC of our model was 0.828$\:\:\pm\:\:$5.28 and 0.818$\:\:\pm\:\:$6.96, while the HDs were 9.80$\:\:\pm\:\:$2.57 and 10.32$\:\:\pm\:\:$1.09.

**Conclusion:**

DL can serve as a surgical guide for beginners and trainees, and can be used as a training tool to enhance surgeons’ surgical skills.

**Supplementary Information:**

The online version contains supplementary material available at 10.1186/s13058-025-01981-3.

## Introduction

Deep learning (DL) has recently been widely used for image classification, detection, and segmentation, particularly in medical image analysis [1]. These DL techniques are used in diagnostic medical imaging to provide speed, efficiency, accuracy, cost-effectiveness, and accessibility in clinical settings [2–4]. In particular, several studies have reported the diagnostic accuracy of DL in breast imaging modalities such as mammography, digital breast tomosynthesis, ultrasound, and magnetic resonance imaging [2].

Recently, active research has been conducted on surgery using DL techniques. Unger et al. used multilayer perception to diagnose and visualize the presence and size of cancerous tumors in breast tissue samples removed during surgery in real time [5]. Shvets et al. developed a technique for automatically segmenting instruments during robotic surgery using U-Net, a network that is commonly used for image segmentation [6]. Lee et al. proposed a method for performing multisurgical instrument tracking in real-world surgeries using a Mask R-CNN to evaluate the surgeon’s performance during surgery [7].

However, studies on image-guided breast surgery to support intraoperative clinical decision making are rare, largely because of the lack of large-scale surgical image data, given that traditional breast surgery is mostly open surgery. Even for endoscopic or robotic breast surgery, no previous studies have applied machine learning to breast surgery procedures and training programs because robotic breast surgery was only introduced a few years ago [8]. Although there are numerous two-dimensional video clips for the education of breast surgery or previous audio-visual material for minimal invasive surgeries for cholecystectomy or gynecological surgery [9, 10], there is currently no available visual guide material based on deep learning for real-time application in the operation field of breast surgery, particularly robotic or endoscopic procedures. Effective training programs for new surgical methods are crucial for novice surgeons [11]. Therefore, we propose a DL-based algorithm that learns the boundaries of the mastectomy cross-section in breast surgery videos to develop a surgical guide as a visual aid for robotic breast surgery, thereby improving the safety and effectiveness of the procedure and supporting clinical practice or training programs.

## Materials and methods

### Data acquisition for robot-assisted nipple-sparing mastectomy (RNSM)

A total of 174 patients underwent RNSM using the da Vinci Si, Xi, or SP system between November 2016 and December 2020 at Severance Hospital. In the 174 patients, ninety-nine patients underwent RNSM using the da Vinci Xi system and 10 patients were randomly selected in the study. Clinicopathological data were collected from patients’ electronic medical records and video clips of the surgery. All patients were informed that a recorded surgical video could be used for academic research and educational purposes and signed a consent form. The video clips do not include any personal information of the patients. The ten videos had a spatial resolution of 1280 × 1024 pixels and a temporal resolution of 30.00 frames per second. Images were obtained by splitting each video into frames at 1-s intervals and converting them into the PNG format. Approximately 20,000 frames were initially extracted. To enhance quality, frames showing operating rooms or containing significant artifacts were manually identified and excluded, reducing the dataset to 8,834 frames. From the 8,834 extracted images, 428 images were selected after excluding images with high noise or without regions of interest (ROIs), and were divided into training, validation, and testing datasets at a ratio of 7:1:2. To ensure the dataset represented a diverse range of video content with minimal duplication, random sampling was employed. The surgical landmarks were marked by two experienced surgeons.

All RNSM procedures were performed by a single experienced breast surgeon with 23 and 10 years of experience in clinical practice and robotic breast surgery, respectively. Briefly, a skin incision was made anterior to the mid-axillary line below the axillary fossa. First, a sentinel lymph node biopsy was performed manually without robotic assistance using monopolar electrocautery or an advanced energy device, such as a bipolar energy vessel sealing device or ultrasonic shears. Second, the retromammary space was dissected, and tumescent solution was injected into the subcutaneous layer. After injecting the tumescent solution for hydrodissection, tunneling in the same layer was performed using Metzenbaum scissors and/or vascular tunnelers. Multiple tunnels were formed along the subcutaneous layer as landmarks for the dissection layer. A single port was inserted into the incision, and the robotic surgical system was docked. After docking, carbon dioxide gas was insufflated through a single port to expand and secure an operating space that included multiple tunnels, and video recording was initiated. The entire dissection of the skin flap was performed using the robotic surgical system. After the procedure, all breast tissues were retrieved from the incision site.

### Ground truth of labeling

**Fig. 1:**
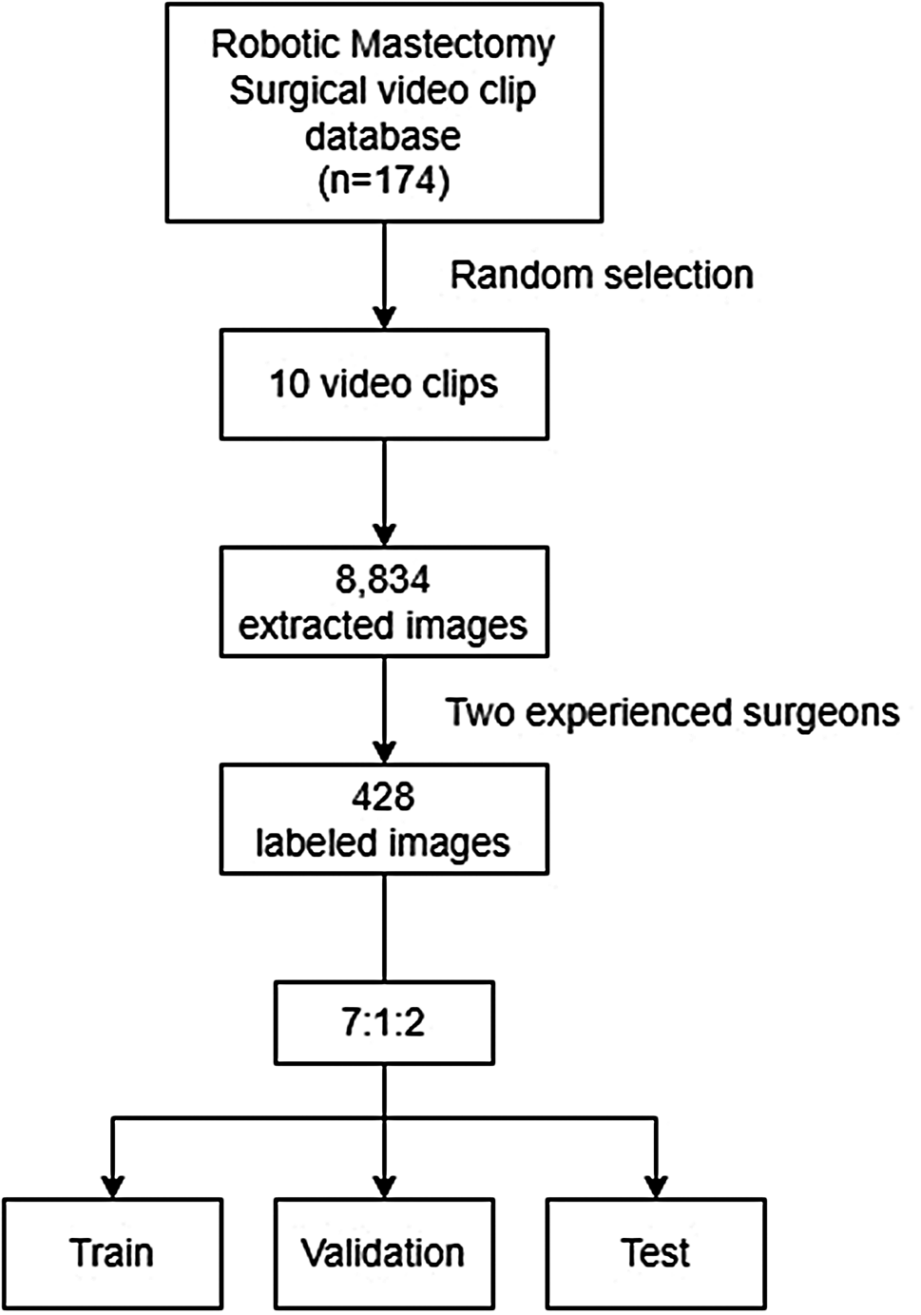
Schematic flow of the current study

Two experienced surgeons marked the tunnels to create a surgical guide to accurately estimate the dissection planes of the skin flaps. One surgeon performed the RNSM procedure, and the other was a breast surgeon with 2 years of experience in clinical practice and robotic breast surgery. Two ground-truth references were created for each image, as each surgeon drew the tunnel manually using the ImageJ software. To achieve surgical guidance, an imaginary line was initially created by connecting the centers of the tunnels. The overall schematic is presented in Fig. 1.

Upon reviewing the initial results, the researchers observed that the imaginary line was closer to the skin flap than to the actual dissection plane. To improve the guide, the imaginary line was revised by connecting the center of the tunnel bottom. The revised results were evaluated by two surgeons who labeled the prediction lines for skin flap dissection.

### DL architecture

**Fig. 2:**
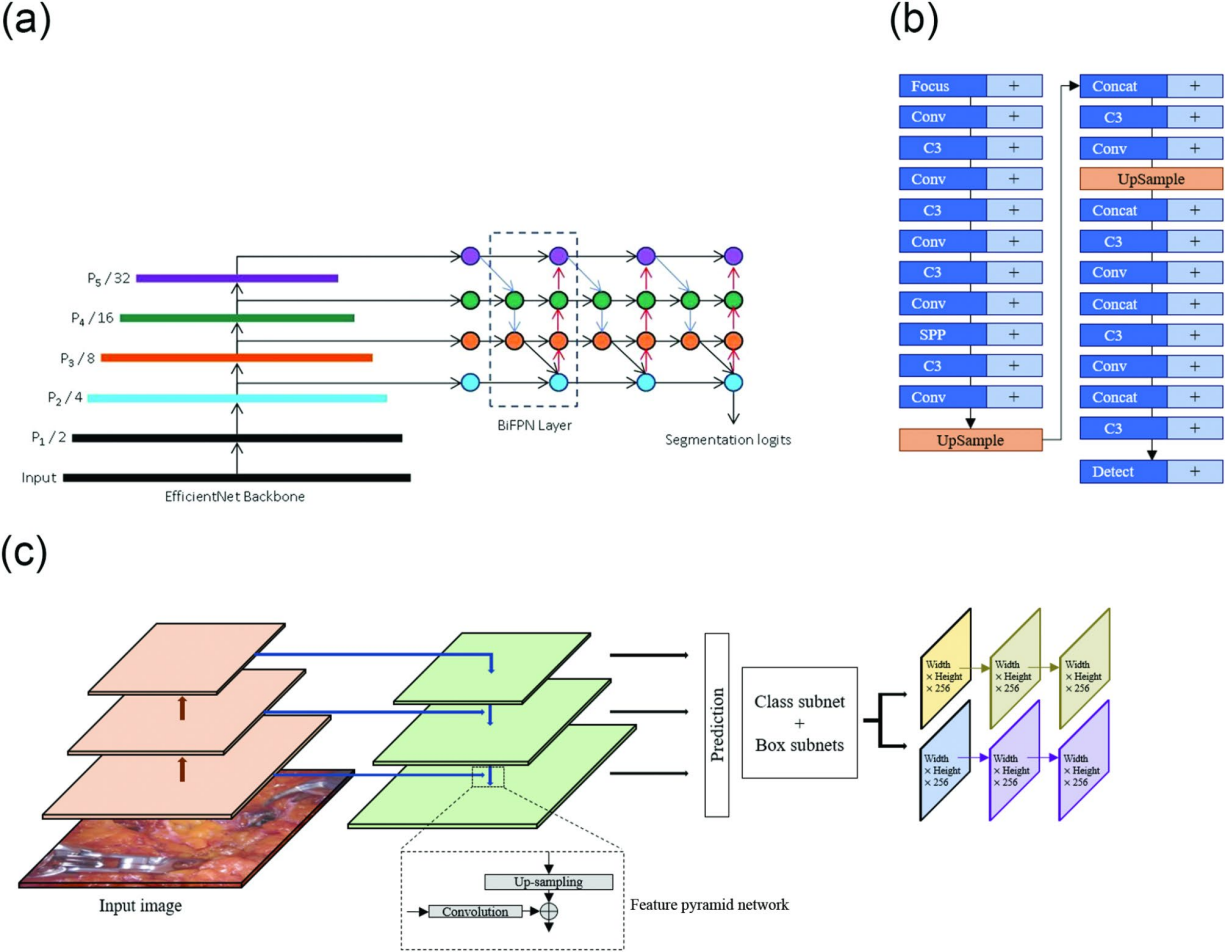
Schematic structures of network architectures. (**a**) Modified EfficientDet. (**b**) YOLO v5. (**c**) RetinaNet

The proposed architectures comprised a modified EfficientDet (mEfficientDet) [12], YOLO v5 [13, 14], and RetinaNet [15]. The proposed architecture consisted of a convolutional neural net (CNN) with a mEfficientDetmodel. A schematic of EfficientDet-b0 is presented in Fig. 2(a). The structure of mEfficientDet uses EfficientNet [12] as the backbone (Fig. 2(a)), and the final structure, which uses a feature network, consists of four layers of BiFPN [16] stacked on top, which is a segmented prediction layer that predicts the target region pixel by pixel. EfficientNet, which is used as the backbone network, consists of several converged layers, MBConv blocks, and converged 1 × 1, pooled, and fully connected (FC) layers. In Conv 3 × 3, one convolutional layer was stacked with a 3 × 3 kernel and 32 channels, followed by one MBConv1 block with a 3 × 3 kernel and 16 channels, two MBConv6 blocks with a 3 × 3 kernel and 24 channels, two MBConv6 blocks with a 3 × 3 kernel and 40 channels, and three MBConv6 blocks with a 3 × 3 kernel and 80 channels. MBConv6 performs depth-wise batch normalization and sweep processes in MBconv. Finally, the FC layer is stacked, encompassing convolutional, pooling, and dense layers, using a 3 × 3 kernel. MBConv uses depth-specific convolutional layers. Unlike regular convolution, which affects all channels, depth-wise convolution partitions the feature map by channel and applies the convolution to only a single channel, which can exponentially reduce the computation. Subsequently, normalization was performed using batch normalization to adjust the mean and standard deviation of all inputs in the batch. A sigmoid-weighted linear unit (switch) was used for activation. A BiFPN is a type of fully convolutional network, where 1 × 1 convolutions act as FC layers. In particular, a BiFPN can be considered as a learning sequence and path through convolution. A segmentation logit is a network that handles the final goal region. The last layer in the BiFPN classifies the target region and processes the final prediction using a layer that handles the boundaries of the region. To supplement the current accuracy, we applied YOLO v5 (Fig. 2(b)) [17, 18], an object-detection algorithm that can detect objects faster with fewer parameters and computations while maintaining high accuracy. This network is based on a backbone that uses a cross-stage partial network that splits the channels, combines multiple small ResNets to create a lightweight structure, and combines focal loss and centered intersection over union (cIoU) loss. The cIoU loss is a loss function designed to estimate the location and size of an object more accurately by improving the IoU. Unlike the conventional IoU, the cIoU loss considers the box’s center point, width, and height to calculate the error. Therefore, using the cIoU loss can improve the accuracy of the object location and size estimation. Moreover, to compare the performance of various state-of-the-art networks, we trained and executed a network called RetinaNet (Fig. 2(c)) [15, 19], which consists of a backbone model composed of a ResNet and feature pyramid network (FPN), and a network for object detection using a new loss function called focal loss. The RetinaNet network can detect objects of various sizes using an anchor box and classification and regression layers, which use feature maps of various resolutions. The model focuses more on difficult examples by increasing their weight using focal loss, and predicts the location and size of objects using Smooth L1 Loss to minimize prediction errors.

### Training of DL models and post-processing

**Fig. 3:**
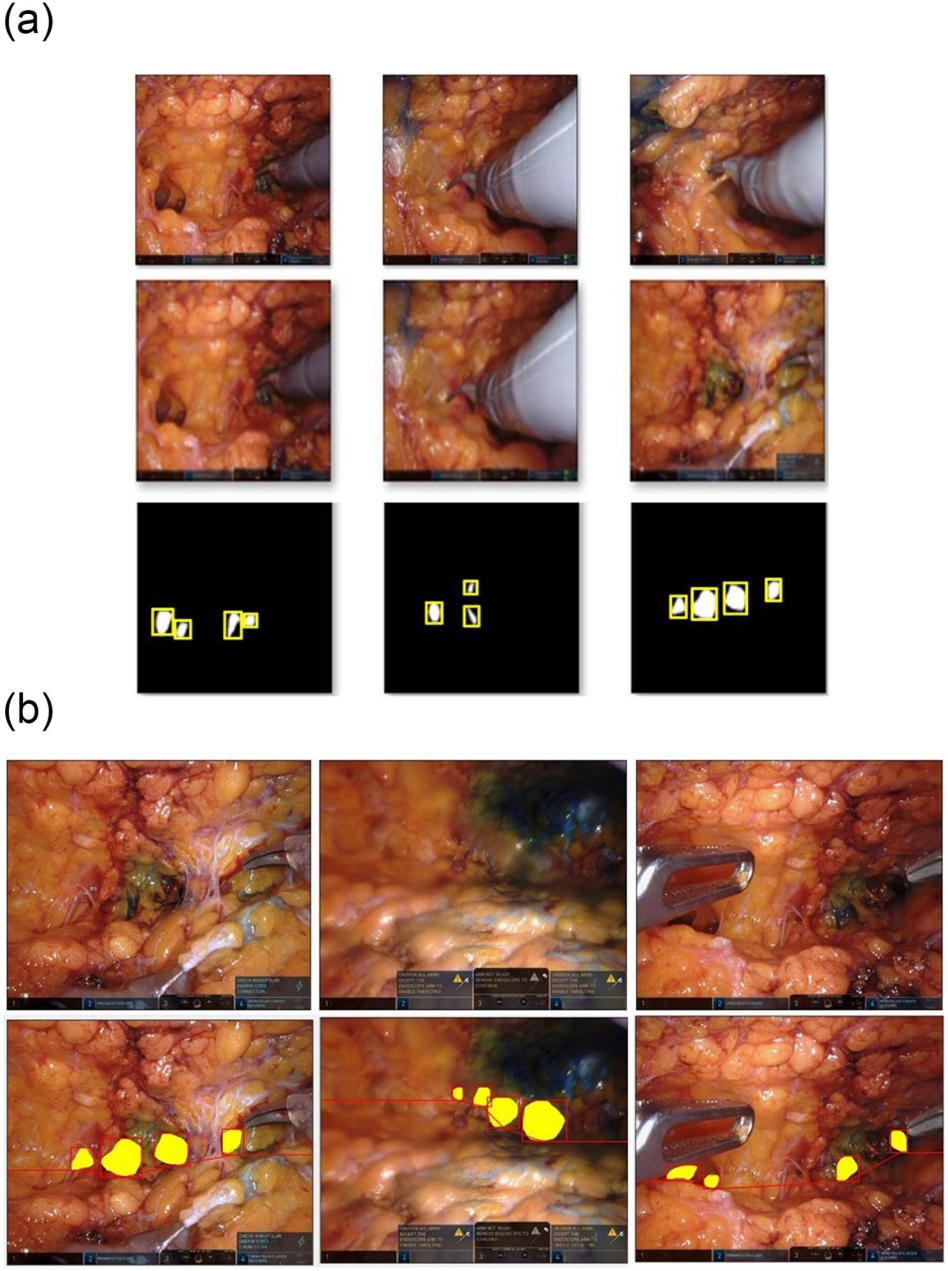
Process of learning the imaginary boxes and lines. (**a**) Generation of the bounding box. (**b**) Imaginary line connecting the boxes and extending the line to both ends of the image

We used 512 × 512 input images for training, applying a normalization method involving mean subtraction and division by the standard deviation. From 8,000 images, 4,072 normal and 428 labeled images were selected. We excluded 3,500 images because of excessive noise, motion artifacts, or low resolution. Subsequently, a normal dataset was added based on the extracted images to split the total dataset into training, validation, and test sets at a ratio of 7:1:2 with no duplicates. To facilitate learning, we generated bounding box labels based on the ground-truth region labels shown in Fig. 3(a). We created two datasets, one with normal images and the other with target regions, because the DL network determines whether an object is inside a box covering a certain region, and because images need to be examined with and without objects.

To augment the data, we randomly flipped the training set both vertically and horizontally. To construct the ensemble model framework, we trained the submodels using the k-fold cross-validation method, considering a small amount of data. We then combined and averaged the prediction results from each submodel using a 5-fold cross-validation procedure. The focus Tversky loss function was used, and the network with the highest validation accuracy after 700 training iterations was selected as the final network. The batch size used for each training iteration was 16, and the learning rate was set to 1e − 4. The default initial learning rate for the network was 0.001, and the network was trained using the Adam optimizer. For YOLO v5, we set the learning rate to 0.001, the number of training epochs to 1000, and the batch size to 16. We also used Adam with an IoU loss as the optimizer. For RetinaNet, we used a learning rate of 0.0001, a batch size of 16, AdamW as the optimizer, and focal loss as the loss function.

For the input image, the training parameters were set using grids [64, 32, 16] and anchor sizes [32, 64, 128]. The anchor ratios were set as [0.5, 1.2]. With approximately 33.5 to 53.1 million parameters and 105 layers, RetinaNet is a network designed to address class imbalance in object detection [15, 20]. The model is based on an FPN that uses two parallel branches to predict object- and class-specific scores at each FPN level; this allows RetinaNet to accurately detect large and small objects regardless of their size. RetinaNet al.so uses focus loss to address class imbalance.

The range for guiding the surgical site is important in robotic endoscopic breast surgery. Therefore, we set the region based on the box. We used the windowing technique to scan the entire image area based on the predicted box and network learning to determine the presence of a tunnel in each box region. We also connected the center points under the box according to expert advice. To evaluate the model, we drew imaginary lines at both ends of the image on the basis of the box (Fig. 3(b)) and connected the center points at both ends of the box. We then connected the lines at both ends of the image outside the box to form a closed area (Fig. 3(b)).

### Evaluation metrics

The evaluation metrics for evaluating the similarities between the predictions of the physicians and the trained model were indicators used in segmentation tasks (e.g., image segmentation), and were measured using the Dice similarity coefficient (DSC) and Hausdorff distance (HD) [21]. The DSC measured the difference between the ground truth and predicted values in video images, returning 1 when the labeled and predicted areas were identical and 0 otherwise. Meanwhile, the HD calculates the error distance for specific pixel values between the ground truth and predicted values, where lower values indicate lower error.

$$ DSC{\rm{ }} = {{2 \times TruePositive} \over \matrix{\left( {TruePositive + FalsePositive} \right) \hfill \cr + \left( {TruePositive + FalseNegative} \right) \hfill \cr} }$$

$${d_H}\left( {X,Y} \right) = \left\{ \matrix{ su{p_{x \in X}}in{f_{y \in Y}}d\left( {x,y} \right), \hfill \cr su{p_{y \in Y}}in{f_{x \in X}}d\left( {x,y} \right) \hfill \cr} \right\}$$

Using the DSC, the predicted results were compared with the guidelines drawn by two experts, each of whom provided guidelines based on the ROI around the target area. Specifically, all ROI object boxes were connected based on the bottom center of the ROI box and extended to the ends of the image to form an area. To validate the annotations for accuracy, we utilized an inter-observer agreement, where two surgeons independently reviewed a subset of the annotated images to assess consistency across annotators. The DSC between these two surgeons was measured to quantitatively evaluate the level of agreement on the annotations, resulting in a DSC of 92.28%.

## Results

The median age of the patients was 47.5 (38–52) years, and the median body mass index was 22.00 (19.30–29.52) kg/m^2^. The median console procedure time was 32 (21–48) min, and the median specimen weight was 352.5 (210–673) g (Table 1). Nine patients had breast cancer, and one patient had a germline *BRCA* mutation. All patients underwent RNSM with immediate breast reconstruction using the da Vinci Xi system. No open conversions, intraoperative complications, breast-cancer-related recurrences, or deaths were observed.

**Table 1:** Characteristics of patients who underwent robotic mastectomy

Variables	Median (min–max) (*N* = 10)
Age (years)	47.5 (38–52)
BMI (kg/m^2^)	22.00 (19.30–29.52)
Console time (min)	32 (21–48)
Specimen weight (g)	352.5 (210–673)

BMI, body mass index

**Fig. 4:**
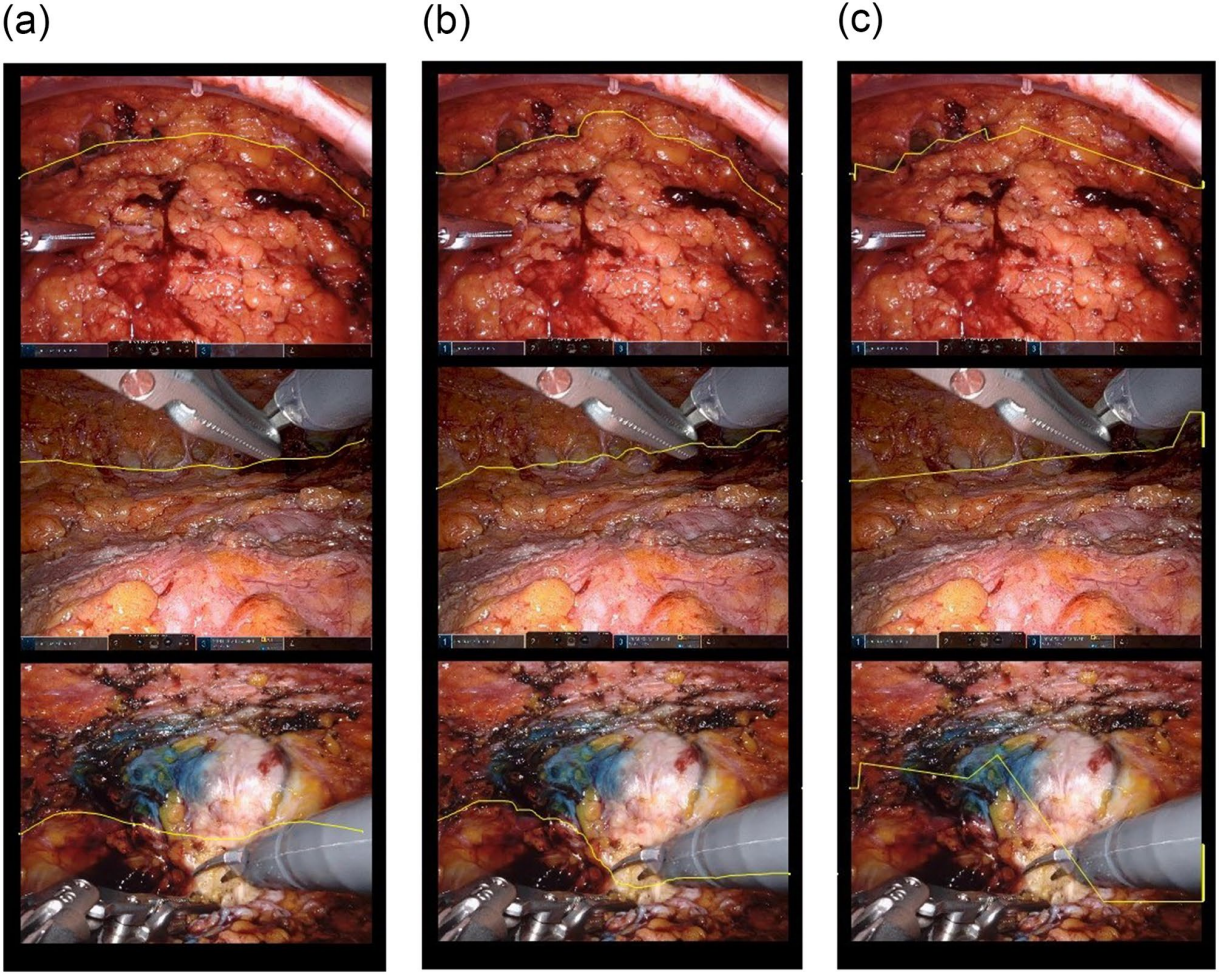
Comparison of the labeling and prediction results of the two experts. (**a**) Expert (1) (**b**) Expert (2) (**c**) Prediction results

When comparing the prediction results Fig. 4(a) and (b) with the areas drawn by the two experts, the average DSC values for the test set were 0.815 and 0.801. Figure 4(c) illustrates an example of the predicted result of the dissection line with the ground truth.

Real-time image analysis was performed by merging the images that were split into the PNG format into videos. The predicted box for the tunnels and imaginary dissection guidelines according to the predicted boxes are presented in the real-time video in *Video*1.

The validation results obtained using the DSC and HD for the three networks are listed in Table 2. In Table 2, DL represents the prediction result of DL, and EXPERT1 and EXPERT2 denote each expert. Among EfficientDet, YOLO v5, and RetinaNet, RetinaNet demonstrated the best results, with DSCs of 82.89$\:\:\pm\:\:$5.28 and 81.88$\:\:\pm\:\:$6.96, and HDs of 9.80$\:\:\pm\:\:$2.57 and 10.32$\:\:\pm\:\:1.09$ for the two experts.

**Table 2:** Results of the validation using dice similarity coefficient and hausdorff distance for the three networks

		DL vs. Expert1	DL vs. Expert2
EfficientDet-b4	Hausdorff distance (mm)	18.84$\:\pm\:$30.39	19.14$\:\pm\:$3.58
	Intersection overlay union (%)	81.50$\:\pm\:$7.71	80.10$\:\pm\:$8.34
YOLO v5	Hausdorff distance (mm)	10.80$\:\pm\:$2.39	11.18$\:\pm\:$1.81
	Intersection overlay union (%)	82.71$\:\pm\:$6.02	81.08$\:\pm\:$8.42
RetinaNet	Hausdorff distance (mm)	9.80$\:\pm\:$2.57	10.32$\:\pm\:$1.09
	Intersection overlay union (%)	82.89$\:\pm\:$5.28	81.88$\:\pm\:$6.96

DL, deep learning

## Discussion

The current study presents the potential application of a CNN with mEfficientDet, YOLO v5, and RetinaNet networks as robotic mastectomy surgical guides for beginners or trainees. The accuracy of the surgical guide in predicting the dissection plane of the skin flap during the console procedure of robotic mastectomy was evaluated by the DSC and HD, and the prediction results of the surgical guide were acceptable.

We also demonstrated that real-time image analysis of robotic breast surgery could be implemented using a video clip (V*ideo 1*). To the best of our knowledge, this is the first report of DL application in robotic mastectomy for developing a visual surgical guide.

In clinical practice, tunneling loosens the subcutaneous tissue before dissection and guides the proper dissection planes. The proper thickness of the skin flap or the presence of residual breast tissue is strongly related to postoperative complications such as skin necrosis and breast cancer recurrence [22–24]. However, during the console procedure, the operator cannot estimate the thickness of the skin flap by touch. Because of the lack of haptic function of the robotic surgical system, a surgical guide for the dissection of the skin flap could improve the postoperative complications and local recurrence rates of breast cancer. In particular, as an effective educational program for beginners or residents, the surgical guide can provide consistent and accurate training not only for RNSM but also for endoscopic surgery. Therefore, it will be possible to apply the results of this study to the education and clinical practice of endoscopic breast surgery, which has been difficult for many surgeons to access easily due to the difficulty in achieving proficiency. While RNSM is in its infancy, previous studies have presented the clinical safety and postoperative outcomes [25–27]. Consequently, this could contribute to the expansion of various breast surgical procedures, education, and improved surgical outcomes.

This study has several limitations. First, we collected the video clips used for developing the surgical guide from a single surgeon, which limits the generalizability of the results. However, because robot-assisted breast surgery is still in its early stages, video clips are lacking. Moreover, because only two skilled surgeons participated in the labeling of the measurement data, more skilled surgeons and the collection of more surgical video clips are necessary. For this reason, we plan to develop a more accurately trained surgical guide using numerous robotic breast surgical videos collected from several experts participating in the prospective cohort study [28]. From a technical perspective, we will apply various state-of-the-art networks developed for box detection to further improve the accuracy of the model. We intend to apply various recent object detection-related networks, such as CenterNet [29, 30], YOLOv7, and Cascade R-CNN, to improve the performance of the DL model used for surgical guide development. Furthermore, we will conduct external validation of the initial model using more videos from a multicenter prospective cohort study and randomized controlled trial in the near future. These further studies will strengthen the implication of the surgical guide for clinical real practice and education.

## Conclusion

These early surgical guides can provide safe and effective training for trainees and novices, leading to reduce the risk of errors and improve the quality of surgical outcomes by providing accurate and reliable guidance during surgery.

## Electronic supplementary material

Below is the link to the electronic supplementary material.

Supplementary Material 1

## Data Availability

No datasets were generated or analysed during the current study.
